# The impact of intimate partner violence on preschool children's peer problems: An analysis of risk and protective factors^☆^

**DOI:** 10.1016/j.chiabu.2015.09.005

**Published:** 2015-12

**Authors:** Erica Bowen

**Affiliations:** Centre for Research in Psychology, Behaviour and Achievement, Coventry University, Priory Street, Coventry CV1 5FB, England, UK

**Keywords:** ALSPAC, Resilience, Domestic violence, Pre-school, Longitudinal

## Abstract

It is unclear whether there is variation in the impact of intimate partner violence (IPV) on child peer problems, and which individual and environmental factors might predict such variation. This study uses data from 7,712 children (3,974, 51.5% boys) aged 4 from the Avon Longitudinal Study of Parents and Children (ALSPAC). Children were cross-categorized based on exposure to IPV from birth to 3 years, and mother-rated peer problems at age 4, into 4 groups: Resilient, Non-resilient, Vulnerable and Competent. Between-group differences in maternal depression, maternal life events, parenting, attachment, and temperament were analyzed, and these variables were also examined as predictors of group membership. Girls were more likely to be identified as resilient. In contrast to the non-resilient group, resilient boys were less emotional, had more secure attachment to their mothers, more interaction with their mothers’ partner, and their mothers reported fewer life events. For girls, the resilient group was less emotional, more sociable, and their mothers reported less depression. Temperament played a stronger role in resilience for girls than boys. There are sex differences in predictors of resilience to IPV within the peer problems outcome domain, which suggests that different approaches to intervention may be needed to foster resilience in boys and girls exposed to IPV.

Intimate partner violence (IPV), defined as physical, sexual and non-physical forms of abuse between current or former intimate partners regardless of frequency or severity (Home Office, 2015), is more likely to occur within married or cohabiting couples that have children (McDonald, Jouriles, Ramisetty-Mikler, Caetano, & Green, 2006). Children under 6 years of age are disproportionately exposed to IPV (Fantuzzo & Fusco, 2007) and yet few studies have examined the impact of IPV exposure on this population (Howell, 2011).

Although substantial evidence documents negative effects of IPV exposure on children, research also shows that a sizeable proportion of children exposed to IPV are not adversely affected by their experience. One meta-analysis of 118 studies found that 37% of children exposed to domestic violence showed no significant developmental problems (Kitzmann, Gaylord, Holt, & Kenny, 2003). The capacity of some children exposed to IPV to develop with no significant problems suggests the existence of individual differences in resilience (Masten & Obradović, 2006). Resilience has been operationalized in different ways in the literature, but the most consistent definition requires that there is evidence of positive adaptation/development in the context of adversity, threat or risk (Luthar et al., 2000, Masten and Obradović, 2006). The methods adopted to assess the intersection between risk and development vary between individual and cumulative risk models (Rutter, 1979). The present article adopted an individual risk model approach through which the contribution of a single risk factor (in this case IPV) to the development of poor child outcomes (peer problems) was examined. This approach has advantages over cumulative risk models as such models provide limited insight into the unique characteristics that lead to protection in the context of risk (Martinez-Torteya, Anne Bogat, Von Eye, & Levendosky, 2009).

The main developmental tasks prior to school attendance concern emotion regulation and prosocial abilities (Graham-Bermann & Follett, 2001). Positive peer relationships developed prior to starting school provide social contexts in which skills essential to children's social, cognitive, communicative, and emotional development are learned and practiced (Guralnick, Neville, Hammond, & Connor, 2007). Children with positive peer relationships also benefit from the sense of affiliation and security that they create, as well as the ability of friendships to mitigate stress. Successful friendships in early childhood contribute to children's quality of life and are important to life adjustment (Overton & Rausch, 2002). Young children exposed to IPV during this period show higher rates of aggression, fighting, hyperactivity and externalizing problems (Margolin, 2005), as well as increased social withdrawal (Howell, 2011). In addition, younger children are more likely to develop poor social competence than are older IPV-exposed children (Rossman, Rea, Graham-Bermann, & Butterfield, 2004). IPV-exposed children are also more likely to experience problematic peer relationships (Katz, Hessler, & Annest, 2007). It is not known whether peer relationships are variably impacted by IPV exposure.

Rates of childhood resilience to IPV vary between 20 and 65% (Graham-Bermann et al., 2009, Grych et al., 2000, Hughes and Luke, 1998, Martinez-Torteya et al., 2009). A number of protective factors have been identified. These include: exposure to lower levels of IPV (Grych et al., 2000), perceiving violence as less serious (Graham-Bermann et al., 2009, Grych et al., 2000), higher social support (Kolbo, 1996), better quality parenting (Kolbo, 1996), low levels of maternal mental health problems (Kolbo, 1996), low depression and PTSD (Graham-Bermann et al., 2009, Howell et al., 2010, Martinez-Torteya et al., 2009), easy child temperament (Martinez-Torteya et al., 2009), and more adept family problem-solving capabilities (Graham-Bermann et al., 2009). Some studies reported sex differences (Kolbo, 1996) whereas in other studies, gender did not account for significant variance (Graham-Bermann et al., 2009, Grych et al., 2000, Howell et al., 2010). In relation to race, those studies that did examine it found no association with resilience (Grych et al., 2000, Howell et al., 2010, Martinez-Torteya et al., 2009). However, all previous studies have been conducted in North America, and it is clear that findings cannot be assumed to generalize to British contexts (Arnett, 2009).

Both the United States and the United Kingdom are Western cultures; however there is evidence that cultural differences can exist between Western countries (Bornstein et al., 1998). Archer (2006) reported that relative to the US, the UK was characterized by lower levels of gender empowerment (a measure of societal gender equality) indicating that there is less gender equality in the UK relative to the US. Gender empowerment in turn, is associated with both the prevalence of IPV and a number of cultural values that govern parenting attitudes and practices. Archer found that gender empowerment was positively associated with measures of individualism, and negatively associated with prevalence of IPV victimization of women. Consequently, the UK was deemed to be less individualistic as a culture than was the US, and have higher rates of IPV. Individualist cultures value self-reliance, autonomy and independence. Parenting goals are to foster these values in children, and authoritative parenting practices (Baumrind, 1971) are typically adopted to this end. Authoritative parents are controlling, demanding, warm, rational, and receptive to children's communication. If we accept that relative to the US, the UK is less individualistic as a culture, it follows that parents will be less authoritative in their interactions with children.

When considering factors that might predict resilience to IPV within the peer relationships domain, it is likely that factors identified in previous studies that have examined internalizing problems as an outcome domain will be relevant (e.g. parenting, maternal mental health, life events). There is evidence from previous studies that risk factors are correlated. Consequently, in-keeping with an individual risk model, the present study used IPV exposure as the index of adversity, rather than a cumulative risk index. However, other risk factors (e.g. maternal depression and maternal life events) that might enhance the negative effect of IPV exposure were also examined. It is equally likely that other factors such as attachment may play a role. Disrupted attachment is a major adverse secondary outcome of IPV exposure (Quinlivan & Evans, 2005). Longitudinal studies have found that when mothers are not sensitively attuned to their children's needs, are less affirming, and more negative toward their infant (styles of relating that women IPV victims are more likely to exhibit than non-victims, Levendosky, Bogat, & Huth-Bocks, 2011), the child develops greater behavioral problems, poor social interactions and more aggressive behaviors (Murray, Fiori-Cowley, Hooper, & Cooper, 1996; Murray et al., 1996).

Martinez-Torteya et al. (2009) observe that research typically adopts a variable-oriented approach to studying the impact of IPV on child development (c.f. Levendosky et al., 2007). However, they counter this approach by arguing that the aggregation of participants into a single group (e.g. IPV exposed children) inadequately represents the individuals within the group. Instead, they suggest that a person-oriented perspective should be adopted, through which behavior is analyzed and understood through predictable patterns that occur across the dependent and independent variables (c.f. Bogat et al., 2005). Taking a person-centered approach to studying resilience enables us to examine and understand specific patterns and associations that exist within groups, through identifying individuals with positive versus negative adaptation (Masten, 2001). In light of this, the four-group model proposed by Masten was used and groups of children were defined in the following ways: (a) resilient children were those who were exposed to IPV but were not rated as showing clinically elevated levels of peer problems; (b) non-resilient children were those exposed to IPV but who were rated as showing clinically elevated levels of peer problems; (c) vulnerable children were those who were not exposed to IPV but who, nevertheless, were rated as exhibiting clinically elevated levels of peer problems; and (d) competent children were neither exposed to IPV, and nor were they rated as having clinical levels of peer problems.

The present study contributes to our understanding of resilience in the context of IPV by drawing on data from a large longitudinal British community cohort, by focusing on the outcome domain of peer problems, by examining risk and protective factors during infancy and pre-school development including attachment, and by analyzing results separately for boys and girls. As it is likely that the IPV experienced by women recruited from clinical settings is more extreme than that experienced more typically by women in community samples (Graham-Bermann et al., 2009) there remains a need to better understand the factors associated with risk and resilience in the context of IPV within community samples. In light of previous research it was expected that a group of IPV-exposed children would be identified as resilient. In addition, IPV-exposed children were expected to have higher levels of peer problems than non-IPV exposed children. Family and child characteristics, specifically attachment, parental involvement, and easy temperament were expected to predict resilience, whereas non-resilience was expected to be predicted by higher levels of maternal depression and maternal life events. Gender differences were expected, and ethnicity and maternal education were included as control variables.

## Method

### Ethics

Ethical approval for the study was obtained from the ALSPAC Law and Ethics committee and the Local Research Ethics Committees.

### Sample

The ALSPAC cohort study (Boyd et al., 2012) aimed to recruit all pregnant women resident in Avon who expected to deliver a child between 1 April 1991 and 31 December 1992; 14,541 women were enrolled (approximately 85% of the eligible pregnant population). These pregnancies resulted in 14,676 known fetuses, of which 14,062 were live births and 13,988 were alive at 1 year. When excluding children born as part of a multiple birth and those who did not survive beyond the first year there were 13,617 mother–child pairs. Data were collected from pregnancy onwards using postal questionnaires. Please note that the study website contains details of all the data that is available through a fully searchable data dictionary (http://www.bris.ac.uk/alspac/researchers/data-access/data-dictionary/).

### Measures

#### Grouping Variables

*Intimate partner violence (IPV)*. Postpartum IPV was assessed at 8, 21 and 33 months. Mothers were asked two questions about whether their partner had been emotionally cruel and/or physically hurt them since the child was born, or referring to the period of the last questionnaire. A woman was considered to have experienced domestic violence at each time point if she responded positively to either physical or emotional cruelty (c.f. Flach et al., 2011). The repeated responses were summarized into a variable identifying those women who had and had not experienced IPV from her partner during the first 33 months of their child's life.

*Child peer problems*. Child peer problems were recorded at 47 months of age using the Strengths and Difficulties Questionnaire (SDQ; Goodman, 1997). Based on community population norms (see http://www.sdqinfo.com/py/sdqinfo/c0.py for details) children were categorized into two groups: average peer problems (approximately 92%; positive adaptation), and high/very high peer problems (approximately 8%; negative adaptation).

#### Protective Factors

*Child temperament*. Mothers completed the Carey Temperament Scales (CTSs; Carey & McDevitt, 1978) at 6 months and 24. Caregivers are presented with a statement describing a certain behavior (e.g. “She lies quietly in the bath”) and asked to rate how often their child behaves in that way on a scale ranging from 1 (almost never) to 6 (almost always). Higher scores indicate more difficult temperament. Two of the nine dimensions of temperament were selected for analysis a priori. ‘Intensity’ and ‘Mood’ were chosen as they correlate most closely with the concept of positive emotionality. The ‘Mood’ subscale is designed to measure the general tone of affect (whether positive or negative overall), and the ‘Intensity’ subscale is designed to capture the level of energy with which an emotional response is made.

At 38 months, mothers completed the Emotionality Activity Sociability (Buss & Plomin, 1984) temperament survey by postal questionnaire. The 20-item survey comprises four subscales corresponding to traits described by Buss and Plomin (1984): emotionality (tendency to show distress), activity (preferred level of activity), shyness (tendency to be inhibited with unfamiliar people), and sociability (tendency to prefer the company of others, Bould, Joinson, Sterne, & Araya, 2013).

*Attachment.* At 42 months post-partum, mothers completed a three-item ‘reunion warmth’ scale the items of which reflect three forms of child behavior that may be present when reunited with mothers after a period of absence. Items are ‘My child avoids me when we are reunited’, ‘My child pushes me away when we are reunited’, and the reverse coded ‘my child wants a hug when we are reunited’. Mothers reported whether these things happened ‘always, sometimes or hardly ever’. High scores are taken to reflect adaptive reunion behaviors theoretically reflective of secure attachment style behaviors (Ainsworth & Bell, 1970).

*Parental involvement.* At 6, 24 and 42 months, mothers reported on their own and their partner's involvement in childcare activities. Nine items were used to determine the frequency (often, sometimes, rarely, never) with which each caregiver engaged in activities such as ‘bathing the child’, ‘feeding the child’, ‘cuddling the child’. High scores reflected more frequent interaction.

#### Risk Factors

*Maternal mental health.* Maternal depressive symptoms were assessed at the same time points as domestic violence using the Edinburgh Postnatal Depression Scale (EPDS, Cox, Holden, & Sagovsky, 1987) a well-established 10-item questionnaire (score 0–30) that has also been validated on non-postpartum women (Cox, Chapman, Murray, & Jones, 1996).

*Stressful life events.* A 42-item Life Event Questionnaire was used. The questionnaire lists a number of events which may have brought changes in mothers’ life. The scale was completed at the same time points as domestic violence. It asks mothers if any of the events occurred since the birth of the child and to indicate the impact on a five point Likert scale: (1) yes, and affected me a lot; (2) yes, moderately affected; (3) yes, mildly affected; (4) yes, but did not affect me, and (5) no, did not happen at all.

*Education and race*. Following the example provided by Flach et al. (2011), maternal education was simplified into a three level variable: low (UK CSE, vocational), medium (CSE at 16) and high (A-levels at 18 or university degree). Due to the high ethnic bias within the sample, participants were categorized as either ‘White or non-White’.

### Analysis

7,712 cases (56.6% of original sample) were analyzed based on complete data, and missing data did not occur at random. The sample reported in this paper were more likely to be non-White (*OR* = 3.09, 95% CI = 2.40–3.99; *χ*^2^ = 83.84, *p* = .000, *d* = .61), were better educated (*V* = .21, *p* = .000; *χ*^2^ = 547.52, *p* = .000) and were slightly less likely to have reported IPV victimization (*OR* = 1.17, 95% CI = 1.03–1.65, *χ*^2^ = 10.37, *p* = 001, *d* = .08). Missing data were deemed not to be missing at random and consequently multiple imputation methods were not considered feasible (Sterne et al., 2009). Therefore analyses were conducted on complete data only.

Where repeated assessment of the same construct using the same measure occurred average scores were used in analyses due to the relative stability over time (*r* = .2–.8). This approach also enabled more parsimonious models to be computed and reduced multicollinearity (c.f. Martinez-Torteya et al., 2009). Odds ratio analyses were conducted to determine the association between IPV exposure and later peer problems. One-way univariate ANOVA were conducted to compare mean scores on each construct (not presented), and Scheffé post hoc tests were used to isolate significant differences. Effect sizes were then calculated where significant differences were identified using Cohen's *d*, where *d* ≤ 0.2 is a small effect, *d* ≤ 50 is a moderate effect and *d* ≥ .80 is a large effect. Logistic regression analyses were conducted with all variables entered together as predictors of resilience versus non-resilient, vulnerable and competent groups. Results were analyzed separately for boys and girls.

## Results

### DV Exposure and the Odds of Resilience

Following the logic of Martinez-Torteya et al. (2009) IPV and peer relationship adaptation were cross-classified to obtain four groups of children: (a) Resilient = exposed to IPV and displayed positive adaptation, (b) Non-Resilient = exposed to IPV and displayed negative adaptation, (c) Vulnerable = never exposed to IPV and displayed negative adaptation, and (d) Competent = not exposed to IPV and displayed positive adaptation. Table 1 presents the frequencies of group membership for the whole sample as well as by gender.

For the sample as a whole the prevalence of IPV experience between birth and 33 months was 17.5%. Overall, 15.2% of the sample was identified as Resilient (14.3% boys; 16.1% girls), 2.3% Non-Resilient (2.4% boys; 2.2% girls), 7.9% Vulnerable (9% boys, 6.7% girls) and 74.6% Competent (74.2% boys, 75% girls). The association between category and sex was significant (*V* = .05, *p* < 0001). The odds of experiencing peer problems were 44% higher in the group exposed to IPV compared to children who were not (*d* = .20). When this association was examined by gender, the odds for boys were 40% (*OR* 1.40, 95% CI 1.10–1.78, *d* = .18), whereas for girls the odds were 52% (*OR* = 1.52, 95% CI 1.17–2.08, *d* = .23). Boys who were exposed to IPV were 40% more likely to be identified as experiencing peer problems than those who were not (*OR* 1.40, 95% CI 1.10–1.78, *d* = .18), and girls were 52% more likely to be identified as experiencing peer problems after exposure to IPV (*OR* = 1.52, 95% CI 1.17–2.08, *d* = .23).

### Protective and Risk Factors

There was no association between resilience category and ethnic group for boys or girls (Table 2, Table 3).

#### Resilient vs. Non-Resilient Children

*Boys.* Non-resilient boys had significantly higher peer problems than resilient boys (*d* = -3.37). Relative to non-resilient boys, resilient boys experienced significantly more frequent interactions with their mother's partner (*d* = .38). In addition, the level of maternal depression reported by mothers of resilient boys was significantly lower than that of non-resilient boy's mothers (*d* = -.40), as was the number of maternal life events (*d* = −.38). Resilient boys were rated as being significantly lower on emotionality (*d* = −.38), and shyness (*d* = −.39) than non-resilient boys, and they exhibited more adaptive behaviors upon reunion with their mothers (*d* = .38). The logistic regression predicting resilient versus non-resilient group membership for boys accounted for only 16% of the variance. Increased emotionality reduced the likelihood of resilience, whereas increased reunion warmth increased the likelihood of resilience, as did frequency of partner–child interaction. Increased maternal life events decreased the likelihood of resilience.

*Girls.* Non-resilient girls had significantly higher peer problems than resilient girls (*d* = −3.40). Resilient girls had mothers who reported significantly lower levels of depression than did the mothers of non-resilient girls (*d* = −.30). In addition, their mothers rated their temperaments as being significantly lower on emotionality (*d* = −.42), less shy (*d* = −.48), and more sociable (*d* = .61) than the temperaments of non-resilient girls. They were also perceived as more active than non-resilient girls (*d* = .37). The logistic regression model accounted for only 12% of the variance. Increased emotionality reduced the likelihood of resilience, whereas increased sociability increased the likelihood of resilience. Increased maternal depression reduced the likelihood that girls would be classified as resilient versus non-resilient.

#### Resilient vs. Vulnerable Children

*Boys.* Vulnerable boys had significantly higher peer problems than resilient boys (*d* = −3.41). In contrast to vulnerable boys, resilient boys experienced less frequent interactions with their mothers partner (*d* = −.46), and their mothers reported higher levels of depression (*d* = .40), and more life events (*d* = .63). When these variables were entered into a logistic regression model predicting resilient versus vulnerable group membership, in combination 39% of the variance was accounted for. The most important individual predictors of resilient versus vulnerable group membership were mother ratings of child interactions with her partner, maternal depression and life events. Specifically, the likelihood of being categorized as resilient decreased as maternal ratings of partner–child interaction increased. In contrast, both increased depression and life events increased the likelihood that boys would be categorized as resilient rather than vulnerable.

*Girls*. Vulnerable girls had significantly higher peer problems than resilient girls (*d* = −3.84). Relative to vulnerable girls, resilient girls had mothers who reported higher levels of depression (*d* = .32) and life events (*d* = .73). In addition, resilient girls experienced less frequent interaction with their mother's partner (*d* = −.52). Resilient girls were rated by their mothers as having less negative mood (*d* = −.21), being less shy (*d* = −.66), and more sociable (*d* = .54) than vulnerable girls. When all variables were entered into a logistic regression model 34% of the variance was accounted for. Six variables were identified as significant independent predictors of resilient versus vulnerable group membership. Higher mood scores decreased the likelihood of resilient group membership as did higher activity scores. Increased distress proneness (emotionality) increased the likelihood of resilient group membership. Increased maternal interaction increased the likelihood of resilient group membership, whereas increased partner interaction decreased the likelihood of this. Finally, girls were more likely to be categorized as resilient as maternal depression scores increased.

#### Resilient vs. Competent Children

*Boys.* Competent boys had higher levels of peer problems than resilient boys (*d* = −.15). There was a significant association between category (resilient vs. competent) and maternal educational level (*χ*^2^ = 8.54, *V* = .05, *p* = .014). Relative to competent boys, resilient boys had mothers who reported higher levels of depression (*d* = .89), and life events (*d* = .89). In addition, resilient boys experienced less frequent interaction with their mother's partner (*d* = −.66). Resilient boys were rated by their mothers as being more prone to distress (*d* = .15), less shy (*d* = −.14), and having more intense moods (*d* = .14). The full regression model predicting resilient versus competent group membership for boys accounted for 35% of the variance. Five significant independent predictors of group membership were identified. High maternal education decreased the likelihood of boys being categorized as resilient. In addition, as mother's ratings of reunion warmth increased the likelihood of being categorized as resilient decreased; this was also the case for maternal ratings of partner–child interaction. Finally, as maternal depression and life events increased, so too did the likelihood of boys being categorized as resilient rather than competent.

*Girls*. Competent girls had higher levels of peer problems than resilient girls (*d* = −.15). In contrast to competent girls, resilient girls had mothers who reported higher levels of depression (*d* = .79) and more life events (*d* = .97). In addition, resilient girls experienced less interaction from both their mothers (*d* = −.14), and their mother's partners (*d* = −.79). Resilient girls were rated by their mothers as exhibiting more intense moods (*d* = .14), and being higher on emotionality (*d* = .20). For girls, the full regression model accounted for 26% of the variance, and five significant independent predictors were identified. As maternal ratings of distress proneness (emotionality) increased, the likelihood of girls being categorized as resilient also increased. Increased ratings of sociability decreased the odds of resilient group membership, as did increased maternal ratings of partner-daughter interaction frequency. Both increased maternal depression and life events increased the odds of girls being categorized as resilient rather than competent.

## Discussion

This is the first study to explore resilience among IPV-exposed preschool children focusing on peer relationships. As expected, a group of resilient children was identified, illustrating the heterogeneous impact of IPV on preschool peer relationships, which parallels its impact on internalizing and externalizing problems more generally (Graham-Bermann et al., 2009, Grych et al., 2000, Hughes and Luke, 1998, Martinez-Torteya et al., 2009). The majority (86.6%) of children exposed to IPV were resilient, supporting the findings of previous studies (e.g. Martinez-Torteya et al., 2009). Resilient children had fewer peer problems than non-resilient groups, and girls were more likely than boys to be identified as resilient (e.g. Martinez-Torteya et al., 2009).

Demographic characteristics did not consistently differentiate groups, whereas individual and family factors did. Consistent with previous studies of resilience to IPV (e.g. Howell et al., 2010, Martinez-Torteya et al., 2009) ethnicity was not found to be relevant to group categorization, although it is acknowledged that this variable was under-specified due to the low frequency of non-White respondents (Boyd et al., 2012). It is therefore possible that ethnic or cultural differences may exist which this study was not sensitive too. Future research needs to examine resilience to IPV in minority ethnic groups as well as bi-racial groups, to ensure that recommendations for interventions to increase childhood resilience are sensitive to cultural and ethnic diversity. For boys, having a mother with a good education differentiated the competent from resilient group only (competent, more likely to be better educated). While some studies suggest that socioeconomic advantage can buffer the impact of risk on child development (Osofsky, 1999) in general, the small number of studies of resilience to IPV during childhood generally fails to support this association (e.g. Kolbo, 1996, Graham-Bermann et al., 2009, Martinez-Torteya et al., 2009). The present findings do little to clarify this association and are difficult to account for.

Consistent with previous research (Graham-Bermann et al., 2009, Grych et al., 2000, Howell et al., 2010, Martinez-Torteya et al., 2009), maternal depression and life events were both implicated in resilience. Children who were exposed to IPV were also more likely to have mothers who reported higher levels of both depression and life events, confirming the correlation between risk factors, and resilient children were exposed to greater levels of risk and protective factors than non-resilient children, which mirrors the findings of previous person-oriented studies (e.g. Martinez-Torteya et al., 2009). Life events were more important in differentiating boys’ resilience-group membership, whereas maternal depression was more important to girls’ membership. Martinez-Torteya et al. (2009) found that the levels of maternal life events experienced by mothers of resilient and non-resilient children did not significantly differ, although the levels reported by those who experienced IPV versus those who did not were significantly different. In the present study, the difference in maternal life events was significant between for the resilient and non-resilient groups, but only for boys. In addition, in the present study, maternal depression differentiated the resilient group from the groups not exposed to IPV but only for boys. For girls, maternal depression differentiated the resilient from non-resilient and competent groups with those exposed to IPV (resilient and non-resilient) also exposed to higher levels of maternal depression than those not exposed to IPV (competent). These subtle variations are difficult to account for. Previous studies suggest a uniform influence of maternal depression with IPV-exposed groups reporting higher levels of maternal depression than non-IPV exposed groups (e.g. Graham-Bermann et al., 2009). In the single previous study to adopt Masten's (2001) four categories of adaptation (Martinez-Torteya et al., 2009) sex differences were not examined. Therefore, it is possible that such differences did exist but were masked. The present study therefore highlights the importance of examining sex differences in relation to the predictors of risk and resilience in the context of IPV. Further research is required that includes the explicit examination of sex differences in order to develop an understanding of the potential consistency of such effects and their meaning.

Family characteristics predicted resilience for boys, whereas for girls, temperament played a greater role. Partner interaction distinguished the resilient group from non-resilient (resilient more interaction), vulnerable (resilient less interaction), and competent (resilient less interaction) groups for boys. Vulnerable and competent girls also experienced more interaction than resilient ones. Previous studies have reported that positive parenting plays a role in resilience to IPV (Kolbo, 1996). These findings show that increased positive interaction with the mother's partner is associated with better child outcomes.

Resilient boys showed more adaptive reunion behaviors than non-resilient boys, but were less adaptive than competent boys, suggesting that resilient boys are more securely attached than non-resilient boys, but less securely attached than competent boys (Ainsworth & Bell, 1970). Non-resilient boys have been raised in violent homes, with mothers who experienced higher levels of depression and life stress; it is likely that their capacity to respond consistently and warmly is compromised (Levendosky et al., 2011). However, this is speculation given that the items were based on child reunion behaviors, rather than parenting style characteristics. Why similar findings for girls were not identified is difficult to account for, but the findings suggest that positive parenting from both partners in the context of risk promotes resilience in boys, and therefore should be targeted through intervention.

Temperament was more relevant to differentiating resilient girls from the other categories than boys, indicating as expected from previous studies that characteristics of ‘easy temperament’ were important for resilience (e.g. Martinez-Torteya et al., 2009). For example, mood and activity independently differentiated resilient from vulnerable girls, with resilient girls having less negative mood, and lower levels of activity than vulnerable girls; characteristics that were not implicated in boys’ resilience. Sociability independently differentiated resilient girls from non-resilient (resilient more sociable) and competent (resilient less sociable) girls. The one characteristic of temperament implicated in resilience for boys and girls was emotionality. Resilient girls were less emotional than non-resilient, but more emotional than vulnerable and competent groups. For boys, emotionality differentiated the resilient group from the non-resilient group (resilient lower), and the competent group (resilient higher). It has been suggested that greater emotional awareness, in combination with physical maturation and language skills make girls more likely to be resilient to problems generally than boys (Zahn-Waxler, Shirtcliff, & Marceau, 2008). These findings highlight the potential role of social skills and emotion regulation training to promote resilience in IPV-exposed preschool children and girls in particular.

There are a number of limitations of this study. Although the strongest regression models accounted for approximately 35% of the variance, it is clear that other, unmeasured variables may also play a role in resilience, and that more expansive modeling is required. For example, researchers have identified empathy and social expressiveness (Luthar et al., 2000), intelligence, locus of control, and self-control (Alvord and Grados, 2005, Masten and Coatsworth, 1998), and self-esteem (Osofsky, 1999) as relevant to resilient development at an individual level. At a family level, although the present study captured parenting and attachment, parental social-competence (Skopp, McDonald, Jouriles, & Rosenfield, 2007) has also been implicated in resilient development. These factors should also be tested within the domain of resilience to IPV.

All variables are based on mother's accounts. Consequently, it is likely that the strength of the regression models is somewhat inflated due to common method variance (Lindell & Whitney, 2001). However, the size of the cohort and age of the children prohibited the collection of independent assessments of behavior and child functioning, although such data would have increased the validity of the findings. Moreover, the children were so young that their own reports would have been unreliable (Martinez-Torteya et al., 2009). It has been found in previous studies based on the ALSPAC cohort that mother ratings of child temperament are not biased by maternal depression (Bould et al., 2013), and nor are associations between temperament and behavioral problem ratings (Stringaris et al., 2010), and so the extent of common method variance in the present study is unclear. It is possible, however, that even if common method variance is low, the reports may have been influenced by social desirability. As mothers were gatekeepers to other respondents, partner reports were only available on a limited subsample and hence excluded from the present study. It is likely that had partner reports been included, the resulting sample would have been even more systematically biased in favor of better-functioning parental relationships, as evidenced by the characteristics of the selective attrition in the sample reported upon herein. It is also unclear from the wording of the questionnaires, whether the ‘partner’ referred to the father of the child. Whilst this is the largest study of preschool resilience in IPV-exposed children, the sample itself is unrepresentative of the geographic region from which it was drawn (Boyd et al., 2012). Finally, the measure of IPV used is limited by its operationalization through only two items. Specifically, defining IPV through items which ask whether partners have been ‘emotionally cruel’ to and/or ‘physically hurt’ mothers might reduce reporting through perceptions that such behaviors lead to particularly damaging psychological and physical consequences. Although more comprehensive measures of this construct exist that include sexual violence and injury (e.g. Conflict Tactics Scale revised, Straus, Hamby, Boney-McCoy, & Sugarman, 1996), items are also included within these measures which represent behaviors that may not be perceived as eliciting the harm inferred by the operationalization of IPV in the present study (e.g. my partner grabbed me). It is recognized that measures which use action-based examples of partner violence lead to higher estimates of partner violence (DeKeseredy, 2000), and through the potential confound of behavior and consequence in the operationalization of IPV in the present study, it is likely that the levels of partner violence reported are lower than those actually experienced, and this is likely to have contributed to the somewhat modest effects found. Although the mothers reported on their experiences of IPV, the extent of children's witnessing is not clear though it is acknowledged that any exposure to IPV can be harmful to children (Fantuzzo & Mohr, 1999).

Despite these limitations, this study is the largest, prospective longitudinal study of resilience in community based pre-school children that have been exposed to IPV. In addition, its focus on peer relationships as the outcome domain makes it unique. The findings suggest some similarities between the factors that promote resilience in this domain, and the broader internalizing and externalizing behavioral problem domains, and further reinforce the need for holistic family-based interventions that increase the quality of parenting and maternal responsiveness to boys in particular. In addition it is clear that women who experience IPV even in the community have mental health needs that require additional support to reduce symptoms, and increase effective coping strategies. Moreover, there is a need for interventions to work with young IPV-exposed girls in order to increase their emotional awareness and regulate their emotional responses. Such interventions will ensure that IPV-exposed children develop healthy, pro-social peer relationships prior to school, which will then reduce the likelihood of longer term peer problems emerging.

## Figures and Tables

**Table 1:** Descriptive statistics by group and gender.

	Non-resilient		Vulnerable		Competent		Resilient	
	Boys	Girls	Boys	Girls	Boys	Girls	Boys	Girls
	*M* (*SD*)	*M* (*SD*)	*M* (*SD*)	*M* (*SD*)	*M* (*SD*)	*M* (*SD*)	*M* (*SD*)	*M* (*SD*)
	*n* = 97	*n* = 81	*n* = 358	*n* = 249	*n* = 2,948	*n* = 2,808	*n* = 571	*n* = 600
Race (% White)	98.2	97.9	98.6	97.3	98.6	98.6	97.1	98.0
Maternal education								
% low	37.2	33.0	27.7	33.7	24.1	23.9	26.9	24.2
% medium	35.4	34.0	34.5	35.0	36.2	34.8	35.3	37.7
% high	27.4	33.0	37.9	31.3	39.7	41.3	37.8	38.1
Mood 6–24 m	17.07 (5.63)	17.53 (6.30)	17.66 (5.24)	18.10 (5.40)	16.21 (4.99)	16.38 (4.95)	16.59 (5.34)	16.98 (5.19)
Intensity 6–24 m	22.51 (5.6)	23.00 (4.22)	22.69 (4.81)	22.42 (4.67)	22.47 (4.77)	22.49 (4.65)	23.15 (4.82)	23.13 (4.57)
Emotionality 38 m	13.94 (4.28)	15.17 (4.49)	13.38 (4.35)	13.88 (4.29)	11.75 (3.94)	12.51 (4.23)	12.36 (4.18)	13.37 (4.29)
Activity 38 m	21.52 (3.67)	20.40 (3.29)	21.03 (3.75)	20.96 (3.28)	21.86 (3.03)	21.40 (3.08)	22.17 (3.00)	21.54 (3.03)
Shyness 38 m	13.15 (4.19)	13.98 (4.69)	13.91 (4.34)	14.76 (4.30)	12.20 (4.01)	12.49 (4.00)	11.63 (3.87)	12.16 (3.78)
Sociability 38 m	17.58 (3.37)	16.93 (3.59)	16.70 (3.83)	17.10 (3.45)	18.14 (2.97)	18.46 (2.91)	18.50 (3.13)	18.76 (2.92)
Reunion warmth	5.37 (1.03)	5.64 (.72)	5.25 (.78)	5.60 (.68)	5.68 (.61)	5.71 (.58)	5.65 (.67)	5.68 (.62)
Maternal parenting	25.04 (3.48)	25.30 (3.57)	25.64 (3.30)	25.44 (3.34)	26.01 (3.02)	26.21 (3.04)	25.77 (3.18)	25.79 (3.36)
Partner parenting	16.62 (7.74)	17.84 (6.74)	21.83 (5.24)	21.57 (5.30)	22.52 (4.93)	22.52 (4.98)	19.10 (6.38)	18.30 (6.70)
Maternal depression	9.42 (4.73)	8.78 (4.32)	6.05 (3.88)	6.19 (4.02)	4.67 (3.42)	4.66 (3.51)	7.69 (4.23)	7.53 (4.23)
Maternal life events	22.47 (8.47)	20.29 (7.12)	15.30 (5.63)	15.11 (6.34)	14.48 (5.46)	14.58 (5.45)	19.59 (7.45)	20.20 (7.29)
Peer problems 47 m	4.66 (.90)	4.49 (.81)	4.71 (1.00)	4.53 (.75)	1.35 (1.08)	1.22 (1.02)	1.19 (1.05)	1.07 (1.03)

**Table 2:** Logistic regression analyses predicting resilience from the alternative adaptation categories for boys.

		Non-resilient	Vulnerable	Competent
		*OR* (95% CI) *n* = 668	*OR* (95% CI) *n* = 455	*OR* (95% CI) *n* = 2,889
Demographics	Race	3.68 (.42–32.06)	.12 (.00–6.54)	.16 (.00–4.24)
Maternal education (low vs. med)	1.20 (.68–2.11)	1.06 (.54–2.10)	.79 (.45–1.40)
Maternal education (low vs. high)	1.66 (.89–3.11)	.82 (.39–1.69)	**.62 (.40–.96)**

Child temperament	Mood	1.02 (.97–1.08)	.96 (.91–1.03)	.97 (.92–1.02)
Intensity	1.01 (.96–1.07)	.96 (.90–1.03)	.99 (.94–1.05)
Emotionality 38 m	.**94 (.89–.99)**	.99 (.93–1.07)	**1.09 (1.03–1.16)**
Activity 38 m	1.03 (.95–1.11)	1.02 (.94–1.12)	.97 (.91–1.06)
Shyness 38 m	.93 (.87–1.00)	.99 (.92–1.08)	1.01 (.94–1.08)
Sociability 38 m	1.05 (.97–1.14)	1.03 (.94–1.13)	.93 (.86–1.02)

Attachment	Reunion warmth	**1.53 (1.18–1.98)**	1.00 (.73–1.37)	**.68 (.52–1.02)**

Parenting	Maternal	1.02 (.95–1.09)	1.04 (.96–1.15)	.98 (.91–10.5)
Partner	**1.04 (1.00–1.08)**	**.89 (.84–.93)**	**.88 (.85–.91)**

Maternal mental health	Maternal depression 8–33 m	.96 (.91–1.02)	**1.08 (1.01–1.17)**	**1.17 (1.11–1.24)**
Maternal life events	Maternal life events 8–33 m	**.96 (.93–.99)**	**1.15 (1.09–1.20)**	**1.13 (1.09–1.17)**

	Model *χ*^2^	60.78, *p* < 001	128.47, *p* < 001	272.29, *p* < 001
	Nagelkerke *R*^2^	.16	.38	.35

*Note*: Boldface type indicates significant associations i.e., the 95% confidence intervals (CI) do not cross 1.

**Table 3:** Logistic regression analyses predicting resilience from the alternative adaptation categories for girls.

		Non-resilient	Vulnerable	Competent
		*OR* (95% CI) *n* = 681	*OR* (95% CI) *n* = 330	*OR* (95% CI) *n* = 2,889
Demographics	Race	.65 (.13–3.17)	4.09 (.44–37.95)	1.84 (.39–8.76)
Maternal Education (low vs. med)	1.40 (.75–2.60)	.99 (.46–12.08)	.79 (.43–1.47)
Maternal education (low vs. high)	1.25 (.66–2.41)	1.18 (.55–2.55)	.66 (.35–1.25)

Child temperament	Mood	1.01 (.96–1.07)	**.93 (.87–.99)**	.96 (.91–1.02)
Intensity	1.00 (.94–1.07)	1.05 (.97–1.13)	1.02 (.96–1.08)
Emotionality 38 m	**.93 (.87–.98)**	**1.09 (1.01–1.18)**	**1.09 (1.03–1.15)**
Activity 38 m	1.02 (.94–1.11)	**.88 (.79–.98)**	.97 (.89–1.06)
Shyness 38 m	.97 (.91–1.05)	.92 (.83–1.00)	1.02 (.96–1.10)
Sociability 38 m	**1.17 (1.06–1.28)**	.93 (.85–1.04)	**.88 (.80–.96)**
Attachment	Reunion warmth	1.12 (.78–1.61)	.97 (.62–1.52)	.82 (.58–1.16)

Parenting	Maternal	.99 (.91–1.07)	**1.10 (1.00–1.21)**	1.03 (.96–1.11)
Partner	1.00 (.97–1.05)	**.86 (.82–.92)**	**.88 (.84–.91)**

Maternal mental health	Maternal depression 8–33 m	**.94 (.88–.99)**	1.07 (.99–1.16)	**1.15 (1.08–1.21)**
Maternal life events	Maternal life events 8–33 m	1.01 (.97–1.05)	**1.10 (1.05–1.16)**	**1.09 (1.03–1.15)**

	Model *χ*^2^	42.92, *p* < 001	84.82, *p* < .001	176.83, *p* < .001
	Nagelkerke *R*^2^	.12	.34	.26

Note: Boldface type indicates significant associations i.e., the 95% confidence intervals (CI) do not cross 1.
